# Supplementary material for: ProteinShader: illustrative rendering of macromolecules
Source: BMC Struct Biol. 2009 Mar 30;9:19. doi: 10.1186/1472-6807-9-19 (PMC2672931; doi:10.1186/1472-6807-9-19)
Supplement: Additional file 1 — ProteinShader program without source code. This compressed file contains the complete ProteinShader program including associated libraries, but no source code. A README.txt file gives an overview of the ProteinShader distribution, and the index.html file in the help subdirectory has directions on getting started with the program as well as a set of tutorials. [file 1472-6807-9-19-S1.zip › ProteinShader-beta-0_9_4-binary/help/tutorials/images/fullsize/1AQB-fullsize.html]

Retinol-Binding Protein


# ProteinShader: Retinol-Binding Protein


♦ Home


♦ Download


♦ Getting Started

Choose a Link:

Installation

Running

Troubleshooting

PDB Files


♦ Tutorials

Choose a Link:

List of Proteins

Style Options

Saving Images


♦ General Controls

Choose a Link:

Menu Bar

Keyboard Shortcuts

Mouse Motion

Motion Panel

Antialiasing


♦ Cartoon Controls

Choose a Link:

Control Panels

Selection Panel

Radio Buttons

Decorations Panel

Adding Textures

Color Panel

Visibility Panel

Side Chains Panel


♦ Atom Controls

Choose a Link:

Control Panels

Selection Panel

Radio Buttons

Color Panel

Visibility Panel

Scale Panel

Tiling Panel


♦ Developers

Choose a Link:

Compiling

Running

Code Docs

Developer Links


Retinol-Binding Protein  
beta-barrel structure  

1AQB tutorial


---

|  |  |  |  |
| --- | --- | --- | --- |
| Hosted by |  |  |  |

Webmaster: Joe Weber,
joe.weber AT alumni.duke.edu
  
Web site last updated: December 30th, 2008
  
© 2007–2008 Joseph R. Weber
